# Could the female-to-male transgender population be donor candidates for uterus transplantation?

**DOI:** 10.4274/tjod.55453

**Published:** 2017-12-30

**Authors:** Murat Api, Ayşen Boza, Mehmet Ceyhan

**Affiliations:** 1 İstanbul Medipol University Faculty of Medicine, Department of Obstetrics and Gynecology, İstanbul, Turkey; 2 American Hospital, Women’s Health Centre Assisted Reproduction Unit, İstanbul, Turkey; 3 University of Health Sciences, Zeynep Kamil Women and Children’s Health Training and Research Hospital, Clinic of Obstetrics and Gynecology, İstanbul, Turkey

**Keywords:** Uterus, transplantation, live donor, transgender people

## Abstract

**Objective:**

To evaluate the eligibility of female-to-male (FtM) transgender people as donor candidates with regard to histologic, surgical, and social aspects.

**Materials and Methods:**

In this prospective cohort study, 31 FtM transgender people underwent standard hysterectomy and bilateral salpingo-oophorectomy for gender reassignment upon their request. The pelvic viscera of the transgender people was intraoperatively observed and the histology of the removed uteri were evaluated for fertility capacity and procurement surgery. A questionnaire was administered to explore their attitude towards uterus donation.

**Results:**

The mean ± standard deviation age was 28.5±5 years. The median duration of testosterone supplementation was 2.4 years; therefore, they all had irregular menstrual periods during this therapy. None had any previous abdominal surgery or additional morbidity. The mean uterine volume was 138±48 cm^3^. No adenomyosis, endometriosis, polyps, adhesions or uterine anomalies were either observed or reported. Endometrial histology was reported as proliferative (58%), atrophic (29%), and secretory (13%) pattern. Of the 31 transgender people, 30 (96.7%) had a positive attitude; only one had no opinion at the beginning. After detailed information about the procedure was given, 26 (84%) still wanted to volunteer for donation, but 4 (12%) changed their opinion to negative (p=0.12, McNemar test).

**Conclusion:**

The proposal of the FtM transgender population as uterus donor is a hypothetical model that has not been experienced before. Nevertheless, our experience revealed that the FtM transgender population would be good candidates socially, legally, and biologically.

## PRECIS:

Female to male transgender population would be good for the candidates socially, legally and biologically.

## INTRODUCTION

Absolute uterine factor infertility (AUFI) is characterized by any condition that causes congenital/iatrogenic absence or non-function of uterus, such as severe intrauterine adhesions or multiple leiomyoma, which may destroy the complete architecture of the uterus^(1,2,3)^. Although congenital absence of the uterus Mayer-Rokitansky-Küster-Hauser (MRKH) syndrome was estimated to be present in 1/4500 female births^(4)^, AUFI affects one in every 500 women of reproductive age^(5)^.

Adoption or gestational surrogacy are the currently available options to overcome childlessness in women with AUFI because an artificial uterus to support the embryo and carry the foetus till birth has not yet been invented. However, one or both of the options are forbidden or are not acceptable in several countries due to social, legal or religious reasons. For example, in Turkey surrogacy and in Egypt adoption are not legally approved. Uterus transplantation (UTx), despite it still being at a very experimental stage, is a reasonable option for fertility achievement. It differs from other organ transplantations with its temporary feature that has been kept until the recipient has delivered the desired number of children to limit the immunosuppression period. Therefore, it brings some important clinical and ethical considerations that need to be addressed. The ethical consideration has already been discussed by Farrell and Falcone^(6)^; therefore, it is beyond the scope of our paper. The clinical issues of this new procedure will be discussed in the context of donor candidates.

The first human UTx was carried out in 2000 in Saudi Arabia^(7)^, with a uterus from a 46-year-old live donor. It was transplanted into a patient who had undergone emergency peripartum hysterectomy during her first childbirth. This attempt resulted in uterine prolapsus and necrosis followed by removal of the transplanted uterus 3 months after transplantation. The second UTx was reported by Ozkan et al.^(8)^ from Turkey in 2013 in a recipient with MRKH syndrome who had undergone previous surgery for vaginal reconstruction. The uterus was procured from a brain-dead donor. Eighteen months after the UTx, the patient underwent two embryo transfer cycles^(9)^. The first cycle resulted in a biochemical pregnancy, and during the second attempt, an intrauterine gestational sac on sonography was confirmed as clinical pregnancy, but it was aborted. In 2014, Brännström et al.^(10)^ initiated the first clinical trial of multiple transplantations, involving nine women who received uteri from live donors. After 6 months, seven uteri remained viable with regular menses and they reported the first successful UTx that resulted in a live birth with a weight of 1775 grams^(11)^. As of now, 11 human UTx have been reported and four healthy babies have been born from the aforementioned trial^(12)^. This report marks an important development to enable live births from women who lack a uterus.

In 2009, the International Federation of Obstetrics and Gynecology (FIGO) Committee^(5)^ reported that it was unethical to remove a uterus for transplantation from young women who had not had the desired number of children. There seems to be an exception to the FIGO committee opinion. Female-to-male (FtM) transgender people, who voluntarily undergo hysterectomy, can be the most appropriate candidates for donation. The aim of this article was to scrutinize FtM transgender people as to whether they could serve as uterus donors, and to explore their attitude towards uterus donation (UD).

## MATERIALS AND METHODS

From March 2014 to November 2015, 31 FtM transgender people underwent hysterectomy and bilateral salpingo-oophorectomy upon their request after all the legal procedures regarding gender reassignment had been completed. Morphologic and histologic eligibility of the removed uteri were evaluated following surgery. The attitudes of the FtM transgender people towards UD were explored by conducting a survey composed of three choices: positive attitude, negative attitude or no opinion. A senior surgeon (MA) interviewed the transgender people and offered the survey before and after giving detailed information about standard hysterectomy and hysterectomy for procurement. The information about the procedures detailed the type of surgery (i.e. laparotomy/laparoscopy), duration of surgery and hospitalization, and potential complications. Written informed consent was obtained from all patients and the institutional review board approved the study. The McNemar test was used to compare the volunteers before and after giving detailed information about the procedure.

## RESULTS

In our cohort of 31 transgender people, the mean ± standard deviation age was 28.5±5 years. The patients were on testosterone therapy (Sustanon 250 mg/month, Schering-Plough) for at least two years. The median duration of testosterone supplementation therapy was 2.4 years; therefore, they all had an irregular menstrual history during this period. None had any previous abdominal surgery or additional morbidity. No adenomyosis, endometriosis, polyps, adhesions or uterine anomalies were observed or reported. Histologic examination revealed that the mean uterine volume was 138±48 cm^3^. Two patients had intramural myomas with a maximum diameter of 2 cm. Endometrial histology was reported as proliferative (58%), atrophic (29%), and secretory (13%) pattern.

Of the 31-transgender people, 30 (96.7%) had positive attitudes; only one had no opinion at the beginning of the survey. After detailed information about the procedure was given, 26 (84%) still wanted to volunteer for donation, but 4 (12%) changed their opinion to negative (p=0.12, McNemar test).

## DISCUSSION

Procurement of a uterus from a live transgender person has some advantages; being young and healthy makes them ideal volunteers for donation. According to the evidence from studies on kidney transplantation, compared with recipients of deceased-donor kidneys, recipients of living-donor kidneys wait less time for transplantation, have a lower risk of rejection, and have better allograft survival and longer life^(13)^. Moreover, the long-term graft survival of kidneys from live donors is superior to that of kidneys from deceased donors, possibly due to the fact that brain death induces organ injury and associated events. Living donors have to undergo extensive health and psychological assessment. Pre-donation procedures would be more rapid than any other living donors because transgender people have already undergone a two-year psychological and physical assessment for gender reassignment before the operation. Farrell and Falcone^(14)^ commented, “Unlike other living-organ donors, who can expect continued organ system function (e.g., renal or hepatic), the uterus donor loses entirely her ability to have children.” This may trigger some regrets about the donation. FtM transgender people are potential donor candidates who fully volunteer for donation and are more likely to have no regrets concerning this decision.

In the first clinical trial of UTx^(10)^, seven of nine donors were close relatives of the recipients (their mothers or sisters) with a mean age of 53±7 years (Table 1). Unrelated living donors are becoming more common in other organ donations^(15)^. Although living donation in related donors have many advantages for overcoming donor-recipient incompatibility, advances in immunosuppressive therapy make the longevity and function of transplanted organs less dependent on the genetic donor-recipient relationship than in the past^(13)^. It seems that finding an unrelated donor will not always be easy. Either an unrelated living donor might need the uterus before the end of the reproductive age or the uterus might be useless as a healthy donor organ after the reproductive period because the uterus is a single organ. In the previously mentioned trial, the donors were selected among related postmenopausal women^(10)^. Previously, the effect of uterine aging on age-related decline in female infertility was studied and it was revealed that age-related reproductive failure was attributable to oocyte quality rather than the age of the uterus^(16,17)^. However, this evidence has come from older but normally menstruating women. Uterine aging may play a role in the reduction of endometrial receptivity, especially in elderly postmenopausal women. This issue needs to be further studied. On the other hand, these donors used combined oral contraceptives for 90 days before procurement to optimize uterine vasculature. This theoretical approach, which may increase the success of the transplantation, can place the donor in jeopardy of thromboembolism. All of these concerns are far from transgender people who are in their reproductive period. However, we can not obviate the fact that the uterus of transgender people has never harbored a pregnancy or has never been proven functional if it is transplanted. Besides, there is a lack of information as to whether androgen treatment affects the pregnancy potential of the uterus. The transgender patients stated that they had irregular menses under the testosterone supplementation, but when the therapy was suspended, their regular menstrual cycles resumed. It seems that the effect of androgen on the endometrium is transient^(18)^. According to our findings, the uteri of transgender people can be regarded as naïve sources with no morbidity. On this basis, transgender people would be considered as unrelated, readily available, young donors for the future.

Brännström et al.^(11)^ developed a national awareness of this new parenthood option UTx in Sweden. In a recent report^(19)^, the publics’ attitude to UTx was examined and UTx was found to be more acceptable than surrogacy (80% vs. 47%, p<0.001). Surrogacy is not allowed in many countries, and also information on the surrogate mother and their families is scarce. In spite of its potential risk, UTx seems to be more reasonable because it provides intrauterine bonding between the mother and the child. In our study, most of the transgender people accepted the idea of being a uterus donor, but they had some doubts about the uterus procurement surgery. According to the Turkish Lesbian Gay Bisexual Trans-sexual Society records, 1500 FtM transgender individuals (1/25.333 female population) exist. We performed 31 gender reassignment operations over a period of 20 months. It has been estimated that 150 MRKH syndrome cases reach their reproductive ages each year. This rough estimation reveals that if all FtM transgender people agree to donate, this would supply a 10-year demand of UTx for the population with MRKH syndrome in Turkey.

UTx surgery entails isolation of the uterus with bilateral, long venous, and arterial vascular pedicles. The complexity of the surgery is mostly related to the extensive dissection of the pelvic sidewalls, which includes dissection of the ureters from their passages over the iliac vessel bifurcations distally to their inlets into the bladder, and dissection of the uterine veins and uterine arteries from their firm attachments to the ureters. For a living donor, this brings some surgical complications including injury to major pelvic organs, life-threatening bleeding, and infection, amongst other problems. In Saudi Arabia^(7)^, the donor’s left ureter was damaged. Furthermore, in Sweden^(10)^, one of nine donors presented with a utero-vaginal fistula. In addition to extensive dissection of the pelvis, it was reported that the donor surgery lasted 10-13 hours^(10)^.

There is a need for a safe and easy alternative because the duration, complexity, and complications of the operation are unacceptably high for a donor. Transplantation surgery has to be more advanced by using the uterus with short pedicles containing the uterine artery and vein. If this hypothetical short pedicle technique could be achieved, the operations could be risk free and shorter for the donors. Another solution to ease the procedure is the use of other vascular anastomosis rather than uterine vessels. Kisu et al.^(20)^ studied experimental surgical technique for UTx for a long time in Japan. Recently, they suggested that the ovarian vein could be used rather than the superficial or deep uterine vein, which were more difficult to dissect^(20)^. This proposal can make transplantation surgery less invasive and safe for donors. Long venous ovarian pedicles already exist because FtM transgender surgery encompasses bilateral salpingo-oophorectomy.

In all our case series, every transgender operation was performed via laparoscopy without any complications or conversion to laparotomy. Although laparoscopy has several advantages over laparotomy, by considering laparoscopic uterus procurement surgery, some questions have to be addressed with this specific issue:

1. It is well known that laparoscopy provides better abdominopelvic exploration; with laparoscope magnification, it enables fine dissection of vessels and pneumoperitoneum itself theoretically assists the development of pelvic avascular spaces. Besides these advantages, what are the limitations of laparoscopy in transplant surgery?

2. If an endoscopic approach would be preferred for the donor, could vascular pedicles be damaged during the extraction of the removed uterus through the vagina?

3. Would laparoscopic surgery be more acceptable for donors compared with laparotomy because of its better cosmetic results?

In the near future, UTx will be performed more commonly and the need for donors will be exceedingly debated.

### Study Limitations

The results of the study are limited to the patients’ characteristics and their attitudes toward UD. There is no information regarding the reasons motivating their decisions. In addition, the cohort is small, which could limit the validity of the study.

## CONCLUSION

The proposal of the FtM transgender population as a uterus donor is a hypothetical model that has not been experienced before. Nevertheless, our experience has revealed that the FtM transgender population would be ideal candidates socially, legally and biologically.

## Figures and Tables

**Table 1:**
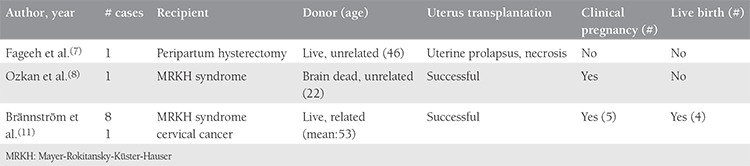
Brief characteristics of previous uterus transplantations

## References

[1] Al-Inany H (2001). Intrauterine adhesions. An update. Acta Obstet Gynecol Scand.

[2] Buttram VC Jr RC, Reiter RC (1981). Uterine leiomyomata: etiology, symptomatology, and management. Fertil Steril.

[3] Grimbizis GF, Gordts S, Di Spiezio Sardo A, Brucker S, De Angelis C, Gergolet M, et al (2013). The ESHRE-ESGE consensus on the classification of female genital tract congenital anomalies. Gynecol Surg.

[4] Folch M, Pigem I, Konje JC (2000). Mullerian agenesis: etiology, diagnosis, and management. Obstet Gynecol Surv.

[5] Milliez J (2009). Uterine transplantation FIGO Committee for the Ethical Aspects of Human Reproduction and Women’s Health. Int J Gynaecol Obstet.

[6] Farrell RM, Falcone T (2015). Uterine transplant: new medical and ethical considerations. Lancet.

[7] Fageeh W, Raffa H, Jabbad H, Marzouki A (2002). Transplantation of the human uterus. Int J Gynaecol Obstet.

[8] Ozkan O, Akar ME, Ozkan O, Erdogan O, Hadimioglu N, Yilmaz M, et al (2013). Preliminary results of the first human uterus transplantation from a multiorgan donor. Fertil Steril.

[9] Erman Akar M, Ozkan O, Aydinuraz B, Dirican K, Cincik M, Mendilcioglu I, et al (2013). Clinical pregnancy after uterus transplantation. Fertil Steril.

[10] Brännström M, Johannesson L, Dahm-Kähler P, Enskog A, Mölne J, Kvarnström N, et al (2014). First clinical uterus transplantation trial: a six-month report. Fertil Steril.

[11] Brännström M, Johannesson L, Bokström H, Kvarnström N, Mölne J, Dahm-Kähler P, et al (2015). Livebirth after uterus transplantation. Lancet.

[12] Brannström M (2015). Uterus transplantation. Curr Opin Organ Transplant.

[13] Reese PP, Boudville N, Garg AX (2015). Living kidney donation: outcomes, ethics, and uncertainty. Lancet.

[14] Farrell RM, Falcone T (2014). Uterine transplantation. Fertil Steril.

[15] Axelrod DA, McCullough KP, Brewer ED, Becker BN, Segev DL, Rao PS (2010). Kidney and pancreas transplantation in the United States, 1999-2008: the changing face of living donation. Am J Transplant.

[16] Navot D, Bergh PA, Williams MA, Garrisi GJ, Guzman I, Sandler B, et al (1991). Poor oocyte quality rather than implantation failure as a cause of age-related decline in female fertility. Lancet.

[17] Noci I, Gheri G, Bryk SG, Sgambati E, Moncini D, Paglierani M, et al (1996). Aging of the human endometrium: peri-implantation phase endometrium does not show any age-dependent variation in lectin binding. Eur J Obstet Gynecol Reprod Biol.

[18] Shufelt CL, Braunstein GD (2009). Safety of testosterone use in women. Maturitas.

[19] Wennberg AL, Rodriguez-Wallberg KA, Milsom I, Brännström M (2016). Attitudes towards new assisted reproductive technologies in Sweden: a survey in women 30-39 years of age. Acta Obstet Gynecol Scand.

[20] Kisu I, Banno K, Mihara M, Hara H, Umene K, Adachi M, et al (2015). A surgical technique using the ovarian vein in non-human primate models of potential living-donor surgery of uterus transplantation. Acta Obstet Gynecol Scand.

